# Health workforce responses to global health initiatives funding: a comparison of Malawi and Zambia

**DOI:** 10.1186/1478-4491-8-19

**Published:** 2010-08-11

**Authors:** Ruairí Brugha, John Kadzandira, Joseph Simbaya, Patrick Dicker, Victor Mwapasa, Aisling Walsh

**Affiliations:** 1 Department of Epidemiology and Public Health Medicine, Division of Population Health Sciences, Royal College of Surgeons in Ireland, Dublin, Ireland; 2 Centre for Social Research, University of Malawi, Zomba, Malawi; 3 Institute of Economic and Social Research, University of Zambia, Lusaka, Zambia; 4 College of Medicine, University of Malawi, Blantyre, Malawi; 5 Department of Global Health Development, Faculty of Public Health and Policy, London School of Hygiene and Tropical Medicine, London, UK

## Abstract

**Background:**

Shortages of health workers are obstacles to utilising global health initiative (GHI) funds effectively in Africa. This paper reports and analyses two countries' health workforce responses during a period of large increases in GHI funds.

**Methods:**

Health facility record reviews were conducted in 52 facilities in Malawi and 39 facilities in Zambia in 2006/07 and 2008; quarterly totals from the last quarter of 2005 to the first quarter of 2008 inclusive in Malawi; and annual totals for 2004 to 2007 inclusive in Zambia. Topic-guided interviews were conducted with facility and district managers in both countries, and with health workers in Malawi.

**Results:**

Facility data confirm significant scale-up in HIV/AIDS service delivery in both countries. In Malawi, this was supported by a large increase in lower trained cadres and only a modest increase in clinical staff numbers. Routine outpatient workload fell in urban facilities, in rural health centres and in facilities not providing antiretroviral treatment (ART), while it increased at district hospitals and in facilities providing ART. In Zambia, total staff and clinical staff numbers stagnated between 2004 and 2007. In rural areas, outpatient workload, which was higher than at urban facilities, increased further. Key informants described the effects of increased workloads in both countries and attributed staff migration from public health facilities to non-government facilities in Zambia to PEPFAR.

**Conclusions:**

Malawi, which received large levels of GHI funding from only the Global Fund, managed to increase facility staff across all levels of the health system: urban, district and rural health facilities, supported by task-shifting to lower trained staff. The more complex GHI arena in Zambia, where both Global Fund and PEPFAR provided large levels of support, may have undermined a coordinated national workforce response to addressing health worker shortages, leading to a less effective response in rural areas.

## Background

Annual funding for the control of HIV/AIDS in resource poor countries rose from $US 1.6 billion in 2001 to $US 10 billion in 2008 [1]. By 2006, an estimated 49% of all external funding disbursed for HIV/AIDS came from two global health initiatives (GHIs) [2]: The Global Fund to Fight AIDS, Tuberculosis and Malaria and the United States President's Emergency Plan for AIDS Relief (PEPFAR). Between 2002 and 2007, the numbers of people on antiretroviral therapy (ART) in developing countries rose from 300,000 to 3 million, leading to a decline in annual AIDS deaths from 2.2 to 2 million [3] and an estimated 550,000 life years saved across 14 African countries [4]. Prevention of Mother to Child Transmission (PMTCT) coverage increased from 9% in 2004 to 33% in 2007 [3]. In some African countries, external HIV/AIDS funding (mainly from GHIs) has exceeded countries' total spend on their health sectors [2], accounting for between 67% and 98% of all AIDS funding in five of the poorest countries [4]. This has fuelled debates about the effects of GHIs on health systems [5]. However, peer-reviewed [6] and other multi-country studies [7,8], until now, have reported mainly national level perspectives, which report contrasting views and expectations of largely positive or negative effects.

The effects of GHIs on countries' health systems is being researched across 16 countries under the umbrella of the Global HIV/AIDS Initiatives Network (GHIN), which supports independent country research teams that have agreed network aims and principles by which they are researching common themes: http://www.ghinet.org. The principal GHIN themes include the effects of GHIs on human resources for health (HRH), on other priority services, on the capacity of countries to coordinate GHIs alongside traditional aid mechanisms, and effects on equitable access to services. Research teams from Malawi and Zambia were among four African country teams and GHIN coordinators who agreed on common research questions, approaches and methods at a research planning workshop in Malawi in September 2006.

Between 2004 and 2008, both countries received large grants from GHIs (see Table 1); and national data illustrate the rapid scale-up in the delivery of HIV/AIDS services (see Table 2). Malawi received large levels of funding from only one GHI (the Global Fund) whereas Zambia received funding from both the Global Fund and PEPFAR. We hypothesised, in conducting the comparative analysis, that it might be easier to roll out a coordinated national human resource for health strategy in a less complex GHI arena. PMTCT services have been rolled out to all 28 districts in Malawi and all 72 districts in Zambia; and nationally reported ART coverage was close to 50% in both countries by 2008 [3]. The World Bank Multi Country AIDS Program (MAP) has also been an external player in funding for HIV in both countries. However, their programme focus was mainly not on health facility scale-up, and therefore was not considered in this paper. This paper presents comparable findings from Malawi and Zambia on the scale-up in service delivery and workload at health facilities, and in numbers and distribution of health workers. The aim is to report trends in health worker numbers, distribution and workload, and to explore and compare the effects of different GHI inputs - Global Fund alone in Malawi and Global Fund and PEPFAR in Zambia - on human resources for health (HRH) strategies and responses, in the light of greatly increased resources for HIV/AIDS.

**Table 1:** Summary of Global Fund and PEPFAR HIV funding to Malawi and Zambia (in million US$)

	Global Fund		PEPFAR^
	**Allocated**	**Disbursed**	**Allocated**

**Malawi**			

Round 1	$342.6 m	$229.6 m (Dec 09)	$14.5 m (2004)

Round 5	$17.6 m	$13.0 m (Oct 09)	$15.2 m (2005)

Round 5 (HSS)*	$ 52.0 m	$21.3 m (Aug 09)	$16.4 m (2006)

Round 8	$15.1 m		$18.9 m (2007)

			$23.9 m (2008)

**Zambia**			

Round 1	$90.3 m	$81.9 m	$82 m (2004)

Round 4	$236.3 m	$128.0 m	$126 m (2005)

Round 8	$129.4 m		$147 m (2006)

			$216 m (2007)

			$269.2 m (2008)

HSS* Health Systems Strengthening

^ Detailed PEPFAR disbursements are not available.

**Table 2:** Core HIV Indicators in Malawi and Zambia

	Malawi				Zambia		
**Indicator**							

	**2004**	**2005**	**2006**	**2007**	**2005**	**2006**	**2007**

Population (in millions)	11.9	12.3	12.8	13.2	11.4^	11.8^	12.2^

Adult HIV prevalence (15-49)% Epidemiological indicators	14.4 (2003)	14.2	No data	12.0	13.9	13.5	13.1^+^

HIV prevalence in pregnant women (%)	19.8	16.9	No data	12.0	19.1	19.1	19.3

Number (%) of adults and children with advanced HIV infection receiving ART	13 183 (6%)	37 840 (14%)	85 200 (33%)	130 488 (43%)	39 351	80 030 (32.9%)	149 199 (50.5%)

Number (%) of pregnant women needing and receiving ART to reduce the risk of mother to child HIV transmission (PMTCT)	2719 (3%)	5076 (7%)	9231 (22%)	23158 (35.4%)	No Data	25,578 29.7%	35,314 39.1%

Women and men 15-49 who received a test in the last 12 months and knew their results.	283 467	482 364	661 400	461 038*	15.6%	234 430 (15.4%)	254 585 (15.4%)

Numbers of sites providing ART	20	60	104	109	107	156	322

Numbers of sites providing PMTCT	36	40	60	84	67	307	678

Numbers of sites providing HIV Counselling and Testing (VCT)	146	239	351	370	No data	883	1028

Source: UNGASS Country Reports 2008

^ Total projected population

^+ ^Zambia Demographic and Health Survey (ZDHS) 2007 shows 14.3% prevalence rate for 2007

* 4^th ^Quarter missing

An analysis of Global Fund proposals [9] and disbursement levels [9], recorded on the Global Fund website, shows that staff training and supplies for Voluntary Counselling and Testing (VCT) and PMTCT were an important component of Zambia's successful 2003 Round 1 US$90 million HIV/AIDS grant. Zambia's late 2005 Round 4 US$236 million HIV/AIDS allocation included a major component of in-service training for 5,264 health professionals and 32,868 non-health agents. US PEPFAR organisations based in Zambia, where US$ 571 million had been allocated by the end of 2007, reported a range of health systems strengthening, infrastructural development and training components. This included the training in 2006 of 'more than 15,000 Zambian health care workers' in the delivery of a range of HIV services [10]. In 2003 Malawi was awarded a large (US$342.6 million) Round 1 grant from the Global Fund to HIV/AIDS control. By 2005 it had re-allocated its grant to support its national Emergency Human Resource Programme [11-13]. The significance of this is considered in the Discussion.

## Methods

### Sampling

Baseline data were collected at district and sub-district facilities in December 2006 - February 2007 and again in June-July 2008. There were common research questions and objectives in the two country studies and standardised tools and indicators were used to research these, with adaptation of questions to suit each country's health information system context. However, both teams had research questions and objectives that were specific to their country, which resulted in different sampling strategies. The Malawi team's main focus was on the effects of HIV service scale-up on health facility staff, for which they derived a nationally representative sample of district and sub-district, urban and rural health facilities. The Zambia team restricted their study to three districts so as to conduct an in-depth analysis of district and sub-district coordination of HIV services, hypothesising that there would be a strong PEPFAR-effect with large-scale utilisation of non-government providers. In Malawi, the districts containing the three tertiary referral hospitals (one from each region) were purposively selected so as to include urban populations; and six out of the 24 rural districts were randomly selected. The 52 facilities sampled included the three central hospitals, seven district government hospitals, and 42 sub-district government health centres. The latter, which represented 30% of district health centres, were randomly selected, with probability of selection proportionate to district facility size, based on a 2005 country-wide survey of HIV and AIDS services [14]. The objective of the Malawi study team was to obtain a representative sample of government health facilities, which were the main providers of ART in Malawi during 2005-08. Non-government organisations (NGOs) and mission (faith-based) facilities were not sampled, as they were not important providers of core HIV/AIDS services in the country.

In Zambia, three districts were purposively selected to represent the capital city (Lusaka), an urban district (Kabwe) and a rural district (Mumbwa). District health facilities were mapped, producing 41 facilities providing fixed HIV or AIDS services. Based on discussions with District Health Management Teams (DHMTs), 39 facilities were selected for the survey (n = 33 government and n = 6 NGO/mission). Facility ART provision was the main criterion for inclusion in the study, and the sample included all 29 facilities that reported delivering ART (24 government and 5 NGO/mission), while excluding Ministry of Defence and private for-profit facilities. The sample also included a purposive sample of 10 facilities that were reported by the DHMTs as important providers of HIV services, though not ART (1 facility in Lusaka, 3 in Kabwe and 6 in Mumbwa). All district, mission and central hospitals, and the University Teaching Hospital (UTH) in Lusaka, were surveyed. The reason for sampling only three districts in Zambia was because a research objective of the Zambian and GHIN researchers was to conduct an in-depth study that explored the roles of non-governmental as well as government providers in HIV scale-up and to assess coordination among providers, in what was known to be a complex provider context. Ethics approval for the study was granted by the University of Zambia Research Ethics Committee and from the College of Medicine in Malawi.

### Data collection tools

Proformas for recording facility record data were drafted by the Dublin GHIN coordination team, adapted from tools used in an earlier SystemWide Effects of the Fund (SWEF) study [7]. These were further adapted, based on lessons learned from a baseline facility survey in Zambia in January 2007. The Malawi team incorporated indicators for measuring scale-up into their tools, which had additional objectives on measuring task-shifting. Semi-structured interview topic guides were drafted by each country team, which included a focus on HRH.

### Surveys, data collection and analysis

Following pilot surveys in both countries, after which further modifications to the data extraction tools were made, trained and supervised teams of field workers visited the selected hospitals and health centres and extracted and recorded facility record data on to the proformas. Facility staff numbers, patient/client records and service episode records covered quarterly periods in Malawi (October 2005 to March 2008) and annual periods in Zambia (2004-2007 inclusive). In Malawi, senior researchers conducted semi-structured interviews with facility frontline health workers (doctors and nurses), facility and human resource managers, and district managers (151), including: facility heads, nurses in-charge of health centres; and district coordinators of ART, VCT and PMTCT. In Zambia, senior researchers conducted semi-structured interviews at the national level (16), including government, donor and NGO representatives. Interviews at the district level (53) were with district health and administration managers, and government and NGO facility managers.

Data on health worker distribution in January 2006 and 2008 that were collected by the research team in Malawi were verified by data provided by district health offices. In Zambia, non-HIV patient record data that were collected by field workers directly from facilities were supplemented by electronic summaries of facility record-return data kept at district health offices. Where there were two sources of data, the most complete data set was used in the analysis. For example district offices had complete data on numbers of Out-Patient Department (OPD) visits from 2004 through to 2007 from 34 of the 39 facilities, compared to 25 facilities whose records' departments had complete data on OPD visits. HIV service data were not available from district offices in Zambia and were collected directly only from the facilities that were delivering ART, VCT or PMTCT.

Quantitative data were entered, cleaned and analysed using SPSS (Version 16.0). Further analysis was conducted using SAS (Version 9.1) to translate data and present findings in similar formats. In Malawi two field workers wrote up contemporaneous notes of interviews, whilst in Zambia, semi-structured interviews were recorded and transcribed. Data coding of different themes was conducted by individual team members and at least two team members undertook thematic analyses [15,16]. Health worker themes included staff categories, numbers, distribution and workload, related to HIV service scale-up.

**Figure 1:**
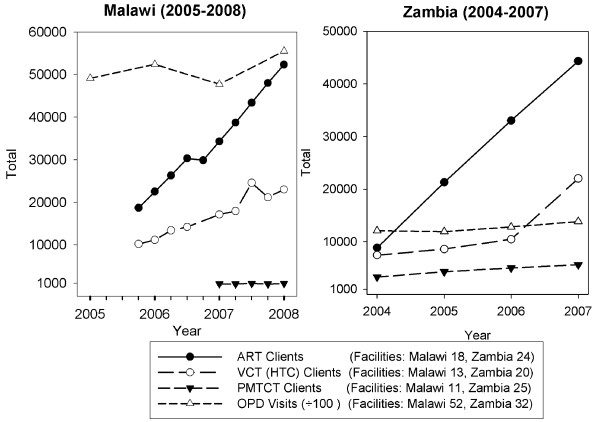
**Scale-up of clients receiving ART, PMTCT, VCT and OPD visits: Malawi (2005-08) Zambia (2004-2007)**.

**Figure 2:**
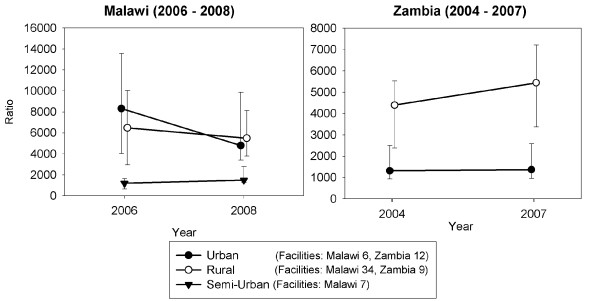
**Urban, semi-urban and rural routine OPD workload per clinical staff member: Malawi (2006-08) Zambia (2004-07)**.

**Figure 3:**
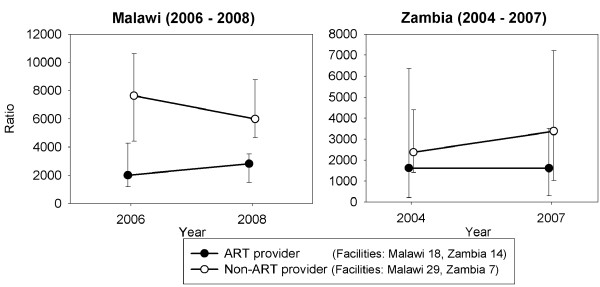
**Routine workload in ART and non-ART providing facilities per clinical staff member: Malawi (2006-08) Zambia (2004-07)**.

Data analysis revealed problems with respect to data availability and completeness, which reduced the numbers of facilities that could be included in some of the analyses. Where facility data were missing for one time period within a trend analysis, this required that that facility be omitted from the analysis, which reduced the numbers of units in some analyses (see Figures 1, 2, and 3). Only facilities that were visited during the December 2006 - February 2007 baseline surveys in both countries were revisited in the follow up surveys (June-July 2008). Therefore, data were not collected from new facilities that opened, or from existing facilities that started to offer HIV related services, during 2007-08. Data cleaning also revealed two implausible records for antenatal clinic registration numbers in Zambia (not part of the analysis for this paper).

## Results

### Trends in scale-up of services: Malawi and Zambia

Figure 1 shows trends in numbers of clients receiving HIV-related services. The numbers of clients on ART and receiving VCT increased consistently over the two time periods in Malawi and Zambia, with similar upward trends across urban and rural districts and at district and sub-district (health centre) levels. In Malawi the 15 month period for which there were PMTCT data showed little increase. This was attributed by national stakeholders to a historical problem with the national collation of PMTCT data, which was the responsibility of a separate section of the Ministry of Health to that collating ART data. In Zambia, there was a steady increase in numbers receiving PMTCT, which almost doubled from 3286 (2004) to 5624 (2007), mainly at urban health centres.

Annual outpatient department (OPD) visits (Figure 1) excluded visits of clients attending for HIV services and women attending for antenatal care or PMTCT in both countries and were used as an indicator of non-HIV routine workload. OPD patient visits were judged to have relied mainly on clinical staff (doctors, nurses and midwives, and clinical officers), who were also responsible for ART service delivery. In Malawi, all 52 facilities surveyed provided OPD care and VCT services, and 29 provided ART. In Zambia, 32 of the 39 facilities reported complete OPD visit data. Six of the other seven, five of which were in Lusaka, were facilities providing HIV related services, such as AIDS care and support, but not routine health services. Twenty six facilities reported ART client data; and 22 reported both ART and OPD visit data. National level respondents in Zambia credited both the Global Fund and PEPFAR for scale-up of HIV services; whereas, at the district level, scale-up was attributed to 'global funds' generally rather than to specific GHIs.

In Malawi, there was a 6% rise in routine outpatient department (OPD) visits, from 5.24 (2006) to 5.56 million (2008). The increase was mainly in semi-urban (district hospital) facilities, where visits increased by 41%, from 0.46 to 0.77 million. In Zambia, there was little change in the numbers of OPD visits, which decreased marginally in urban areas, from 654,132 (2004) to 635,020 (2007) and increased in the rural facilities from 84,229 to 91,444. The higher ratio of OPD to ART clients in Malawi, compared to Zambia, is because a higher proportion of Malawi's large general government health facilities were surveyed, capturing a higher proportion of Malawi's OPD as well as its ART client numbers. In Zambia, most ART scale up was in Lusaka, especially in the University Teaching Hospital and four faith-based clinics, which had a higher ratio of ART to OPD clients compared to Malawi. Lusaka accounted in 2004 for 96% of the ART clients across the three districts in this study, falling to 90% by 2007. The Lusaka ART client numbers, reported in our study, accounted for 54% of *all *ART clients reported by Zambia for 2005, falling to 30% of Zambia's population on ART by 2007 [17].

### Numbers and categories of health workers

#### Malawi

In Malawi, between December 2006 and June 2008, there was a modest (10%) rise in clinical staff (doctors, nurses/nurse-midwives, clinical officers and medical assistants), 127 of 140 (91%) of which were allocated to facilities providing ART (Table 3). Much of the increase was in nurses, whose numbers increased by 13%. There was a larger (81%) increase in laboratory and pharmacy staff, all in urban and semi-urban (district hospital) facilities. Health Surveillance Assistants (HSAs), who were responsible for supporting community Primary Health Care service delivery and had been retrained to support HIV counselling, accounted for three quarters of the 33% rise in all health facility staff. Most of the increase in HSA numbers was in rural health centres where 58% of HSAs were located by 2008.

**Table 3:** Trends in numbers of facility health staff in Malawi (52 facilities) and Zambia (27 facilities): baseline and follow-up ^1^

	Malawi:								Zambia:					
**Health worker category**	**Mar 2006**	**Mar 2008**	**Mar 2006**	**Mar 2008**	**Mar 2006**	**Mar 2008**	**Mar 2006**	**Mar 2008**	**2004**	**2007**	**2004**	**2007**	**2004**	**2007**

	**Urban**	**Urban**	**Rural**	**Rural**	**Semi-urban**^**2**^	**Semi-urban**	**Total**	**Total**	**Urban**	**Urban**	**Rural**	**Rural**	**Total**	**Total**

**Doctors**^**3**^	59	65	2	5	8	10	***69***	***80***	16	23	6	6	***22***	***29***

**Nurses**^**4**^	523	651	221	199	295	329	***1039***	***1179***	384	381	61	61	***445***	***442***

**Clinical Officers & Medical Assistants **^**5**^	135	94	67	85	103	115	***305***	***294***	76	67	16	15	***92***	***82***

**Total doctors, nurses, clinical officers, medical assistants**	**717**	**810**	**290**	**289**	***406***	***454***	***1413***	***1553***	**476**	**471**	**83**	**82**	***559***	***553***

**Technicians**^**6**^	37	65	1	1	24	46	***62***	***112***	51	62	4	11	***55***	***73***

***Health Surveillance Assistants + Dedicated HIV counsellors***^7^	74	158	456	737	205	381	***735***	***1276***	47	56	16	21	***63***	***77***

**TOTAL**	828	1033	747	1027	635	881	***2210***	***2941***	574	589	103	114	***677***	***703***

^1^Numbers of each category of health worker shown are for facilities reporting such staff at baseline and follow-up

^2^The term semi-urban area has been used here to denote district capitals (district hospitals). Rural in Malawi refers to rural health centres. Urban refers to the three main urban centres where the central hospitals and urban health centres are located

^3^Doctors include general and specialist doctors

^4^Nurses include all categories of nurses, midwives and nurse technicians

^5^Malawi: Clinical Officers and Medical Assistants. Zambia does not have a medical assistant cadre

^6^Technicians include laboratory technicians and assistants; and pharmacy technicians and assistants

^7^Health Surveillance Assistants exist only in Malawi only. Dedicated HIV counsellors are reported for both Malawi and Zambia

#### Zambia

In Zambia, between 2004 and 2007, total numbers of health staff increased only slightly (by 4%), from 677 to 703, and numbers of clinical staff remained virtually static (Table 3). Technical support staff (laboratory and pharmacy technicians) increased from 55 to 73 and numbers of dedicated HIV counsellors only increased from 63 to 77. Between 2004 and 2007, clinical staff numbers remained stagnant in both rural facilities (falling from 83 to 82) and urban facilities (falling from 476 to 471).

### HIV and non-HIV workload

Figure 2 shows trends in the average (median) ratios of non-HIV OPD visits to numbers of facility clinical staff in surveyed facilities across the two time periods. Medians are used instead of means to reflect the effects of changes in small as well as large facilities, as changes in facilities with very large numbers of OPD visits can have a disproportionate effect on overall mean staff-patient ratios. Where trends in median and mean ratios diverged, these differences are presented.

#### Malawi

In Malawi, there was a 24% increase between 2006 and 2008 in median OPD workload in semi-urban district hospitals, though rising from a low baseline of 1202 to 1493 patient visits per clinical staff member (Figure 2). There was twice as fast an increase (51%) in the overall mean patient-staff ratio at district hospitals. Median OPD workload reduced from higher levels in both rural health centres (from 6483 to 5574 visits per staff member) and in urban hospitals and clinics (8325 to 4793). However, the overall mean workload remained around 4000 visits per staff member in rural health centres and fell only slightly from 5216 to 4561 in urban facilities. Across the 52 facilities surveyed, the increase in clinical staff and OPD patient visit numbers were comparable so that there was little overall change in workload.

Figure 3 shows a similar analysis of workload, comparing facilities providing ART with those not providing ART. Rural health centres constituted almost all (28 of 29) of the non-ART providers, where workload was measured, so that the downward trend in workload corresponds closely with the downward rural trend shown in Figure 2. The upward trends in non-HIV workload in ART providing facilities in Malawi were from a low base and were found in six rural health centres (rising from 2024 to 2709 OPD visits per staff member) and in the seven district hospitals (1202 to 1493 - see above). In summary, the data show higher routine workloads for clinical staff in rural non-ART providing health centres; and low but rising workloads in all facilities that were providing ART.

Facility managers in Malawi reported that staff numbers had increased, but not at the rate of increase in work-load due to HIV/AIDS service scale-up. The provision of new services, such as nutritional support alongside ART services, had resulted in increased patient attendances, workload and client waiting times due to staff shortages. There were other examples:

*".... The procurement of the CD4 machine has made our workload even worse because everybody in town wants to prove their HIV status here ......... the fact that soon we will be doing viral loads will stress us more if no additional laboratory staff will be recruited*" -

(Hospital laboratory technician, Malawi)

District nursing officers stated that nurses were the most overburdened because they provided most direct care to patients, as well as delivering HIV/AIDS services. Some respondents believed that this was impairing quality of care (though this study did attempt to substantiate this view):

*"... Although the nurses have the skills necessary to counsel a client, they are still following short cuts when executing their duties because of too much work ...... this is so because counselling takes more time to complete and with many clients waiting for you outside, you just do what you can afford......" *(District Nursing Officer, Malawi)

Other respondents believed that service quality was being maintained and that contrary views were more an expression of frustration due to work overload than to actual deteriorations in care. Staff training was reported as a positive effect, in that general care for non-HIV as well as HIV services had improved. By mid 2008, newly trained HSAs in Malawi were providing VCT, reducing the need for clinical staff to allocate time to these activities, especially in district hospitals and health centres. Also, the opening of more sub-district facilities was reported to be reducing client numbers at district and central hospitals.

Facility managers reported that workload, which had been a long-standing and worsening problem in Malawi, was being tackled in several ways, including: training and rotating additional clinical staff through HIV/AIDS clinics, thereby increasing the pool of trained staff and reducing the risk of 'burn-out'. Burnout was more likely if facilities relied on a small number of dedicated staff for delivering HIV/AIDS care. Other strategies included training HSAs, volunteers and retired nurses to provide VCT; integrating PMTCT into routine antenatal care and delivering it after antenatal clinics closed; and paying staff a Global Fund-supported over-time allowance. However, the latter was criticised by laboratory technicians, HSAs and ward attendants who were excluded from the increment and felt it discriminatory when they also worked additional hours.

#### Zambia

In Zambia, routine non-HIV OPD workload, which was already more than three times higher in rural facilities, rose by 24% (from 4397 to 5439 patient visits per clinical staff member - Figure 2), whereas urban OPD workload increased only slightly (from a median of 1319 to 1371). Mean workloads also rose in rural areas, but were only around 20% (18-21%) of the median workloads, principally because the 46-48 clinical staff in Mumbwa district hospital represented around 60% of all clinical staff across the nine rural facilities that were included in the analysis. If this rural district hospital, which appeared to be relatively well staffed and had much lower patient-staff workload ratios, is excluded from the analysis, the mean workloads are twice as high in the remaining rural facilities and the median workload shows a 35% increase over the 2004-07 time period. These findings illustrate the importance of using medians as well as means to measure average workload in samples that include a small number of large and many small facilities.

The analysis of workload (Figure 3) comparing ART and non-ART providing facilities in Zambia suggests that routine workload increased in facilities that did not provide ART, rising from a median of 2380 in 2004 to 3381 OPD visits per clinical staff member in 2007. However, the analysis was based on only seven facilities and the mean workload fell slightly in these non-ART providing facilities. Stratified analysis showed that the increase in mean and median workload, the latter up by 40%, was in the four *rural *facilities that did not provide ART and both measures showed a decrease in workload in the three urban facilities. Mean and median workloads also increased greatly in the five rural facilities providing ART, with the median workload increasing by over 80%, from 3001 to 5439 OPD visits per clinical staff member. In summary, the data show a persistent upward trend in both median and mean rural facility OPD workloads between 2004 and 2007.

Respondents in Zambia reported that voluntary lay counsellors were relieving some of the HIV counselling burden on health staff and that the biggest obstacle now was the shortage of frontline clinical staff (nurses, clinical officers and doctors), especially in rural areas. One district informant commented that due to the significant shortage of staff, it was common for one nurse to attend to up to sixty patients in a ward at a time. Informants in rural Mumbwa, in Zambia, attributed increases in staff workload to the scale-up of HIV/AIDS services coupled with the fact that there had been no corresponding increases in the numbers of staff brought into the health system.

Rural facilities were having difficulty attracting health staff due to a lack of accommodation, despite the rural retention programme [18], introduced as a pilot in 2003, which aimed to retain health workers through the provision of a hardship allowance, housing rehabilitation and vehicle loans. A lack of existing accommodation was mentioned as one reason for the scheme's failure. Several respondents spoke of rural health centres that had only one nurse or clinical officer who was rolling out VCT and ART services in addition to routine duties.

*"... Let's take the rural health centre, where we have only 3 staff they also have to do all this extra paper work, follow-ups etc, so in the end the people are overworked ... No new staff have been brought to the system since these HIV programmes were introduced"*.

(Hospital manager, Mumbwa rural district, Zambia)

During Round Two follow up field work, Mumbwa's district health team was piloting an initiative to encourage school-leavers to take up nursing training and then return to work in the district. The inability to retain staff in Zambia was seen as a financial issue and there were frequent references to higher salaries being offered by PEPFAR-funded NGOs, which were attracting staff away from government service.

*"The biggest problem is like where they have been also providing support to the NGOs and NGOs tend to offer good salaries and health workers (when) trained go to the private sector. The support ... has contributed to brain drain, work overload for the remaining staff"*.

(Donor, national level Zambia)

Where available, population catchment data were collected from district offices in Zambia and from the national level in Zambia to compute and demonstrate trends in clinical staff densities, i.e. the ratios of health facility clinical staff numbers (doctors, nurses and clinical officers/medical assistants) to health facility catchment population sizes, adjusted for population growth. Both sets of data (staff numbers and catchment populations) were available in 36 facilities in Malawi and 18 facilities in Zambia. In Malawi between 2006 and 2008, health worker densities fell slightly in rural health centres from 1.8 to 1.7 per 10,000 and in surveyed urban health centres from 1.7 to 1.25 per 10.000. In Zambia, clinical staff densities in surveyed rural facilities fell from 2.9 (2004) to 2.1 (2007) per 10,000. In contrast, clinical staff densities increased in the urban areas from 6.0 to 7.0 per 10,000, rising from a two-fold to a three-fold greater staff density in urban versus rural areas.

## Discussion

These findings add to the 'thin and contested ... knowledge base' around the effects of GHIs on countries' health systems [19]. Data collected directly from facilities and district offices corresponded with nationally reported data [17,20], confirming that population-wide scale-up of ART, PMTCT and VCT services has been happening in Malawi (2006-08) and Zambia (2004-07). More importantly, it provides facility level data that demonstrate large increases in HIV service client loads, including an almost threefold increase in ART clients over 30 months in Malawi, and a fourfold increase in ART clients over 48 months in Zambia. The type of intra-facility analysis conducted in this study has been able to demonstrate the correlations in trends between ART scale-up, routine workload and the availability of clinical staff at the facility level. While OPD visits provide only one measure of clinical staff workload, they represent an indicator that was routinely reported by facilities to District Health Management Teams. Such evidence therefore does not rely on special data collection exercises.

In Malawi, there was a modest (10%) increase in clinical staff numbers (doctors, nurses and midwives, and clinical officers and medical assistants) at district hospitals and urban health centres, but not in rural health centres where the increase in staff was principally through non-clinical HSAs. The increase in routine workload in facilities providing ART, notably at the district hospitals but also at rural health centres, suggests a steady increase in client utilisation of these facilities. Whether Malawi's decision to allocate most (91%) of the increases in clinical staff to ART facilities was in response to the increased workload, and/or the greater availability of staff helped to attract more patients, it suggests a coherent approach to health worker distribution when faced with the challenge of delivering ART on top of routine care. The increase in clinical staff in Malawi resulted in a decrease in OPD workload in rural and urban facilities, with a slight increase in semi-urban (district hospital) facilities.

ART scale-up in these three districts of Zambia between 2004 and 2007, was set against a static urban routine outpatient workload, a 24% increase in workload in rural facilities and a 35% rise in smaller rural facilities. A recent study [21] reported workload as the most important cause of health worker burnout in urban health facilities. These facilities experienced a net decrease in clinical staff numbers, which was proportionately greater in the rural district, and only a modest increase in support staff (technicians and dedicated HIV counsellors). In 2004, rural Mumbwa facility staff were coping with four times as many OPD visits as Lusaka (the capital city) facilities and twice as many as facilities in urban Kabwe. By the end of 2007, dedicated HIV counsellors in Zambia still only accounted for 11% of staff directly delivering a service to clients/patients in surveyed facilities, compared to counsellors and HSAs in Malawi who accounted for 43% of such staff. Unlike Malawi, these district facilities in Zambia did not appear to be using task shifting to non-clinical staff to manage the increased HIV workload during this period. While there was an upward trend in non-HIV workload in ART providing facilities, which may mean they were attracting more patients, the urban-rural disparity was stronger.

The GHIs, notably Global Fund in both countries and PEPFAR in Zambia, were clearly providing the significant proportion of the external funding which was achieving this impressive scale-up in life-saving HIV/AIDS service coverage. An increase from US$3 (2003) to US$5 (2006) per capita expenditure on HIV in Malawi and from US$10 to US$14 per capita in Zambia was due to external resources [4]. The perception at the national level in Zambia was that in 2008-09 PEPFAR would account for half and the Global Fund for one third of all funding for ART roll-out [22]. Several reports and other studies have pointed to a large and longstanding degree of rural-urban inequity in Zambia. Only 52% of all health workers and 24% of doctors live and work in rural areas where two thirds of Zambians reside [23], and there are high vacancy rates and a rapid turnover of staff in rural areas [24]. Zambia's Public Expenditure Review national HRH survey [25] reported much higher vacancy rates in rural compared to urban health centres for the following health worker categories: doctors (91%:38%), clinical officers (58%:43%), midwives (50%:32%), nurses (43%:23%). Attribution of findings on health workforce distribution, trends and incentives to the inputs and influence of the Global Fund and PEPFAR - and to government responses to GHIs - is more difficult. However, the findings from this study show a divergence and a deterioration in rural-urban equity in Zambia, during the period when PEPFAR and the Global Fund were likely to be having a major impact.

WHO specifies a minimum workforce threshold estimate of 2.28 clinical staff (doctors, nurses, midwives) per 1,000 people [26] (23 per 10,000). Clinical staff densities in our study (between 2.9 and 2.1 in the rural facilities and between 6 and 7 in urban facilities) were lower than the 7.9 per 10,000 that have been reported nationally in Zambia in 2004 which had risen to 9.8 per 10,000 in 2007 [23]. This could partly be attributed to lack of designated catchment populations for the large district and central hospitals. The University Teaching Hospital did not provide data on staff numbers. Rural Mumbwa district (at 2.9 in 2004 falling to 2.1 in 2007), however, was typical of health worker densities in three of six rural districts cited in an early draft of the Global Fund's Five Year Evaluation [4], which were categorised as 'poor infrastructure rural' (mean 2.6, range 1.7-3.5). More weight can be given to the Zambian than to the Malawi staff density findings, as in the former all public and private fixed facilities were mapped and were included in the study if they were providing ART. In Malawi, only public sector and faith-based facilities were included, which meant that clinical staff in NGO facilities, likely to be common in urban areas, were not included in the study.

The slightly larger rural-urban difference in nationally reported health worker density in Zambia (4.5:16.0) [23], compared to Malawi (3.5:11.7) [27], may reflect contextual differences: an estimated 35% of Zambia's population live in urban areas [28], compared to 18% in Malawi [29]. The population density in rural areas of Malawi is six times that of Zambia and is among the highest rural densities in the world [30]. However, whatever the underlying factors, the evidence (based on one rural district) suggests that some rural areas have been falling behind urban areas in Zambia in terms of clinical staff allocations, during the period that GHI funded scale-up accelerated. While this study did not aim to measure rural-urban ART coverage levels, the high proportion of Zambia's nationally reported ART client estimates that were attending facilities in Lusaka suggests that ART service scale-up was heavily skewed towards the capital city, at least during the 2004-07 period.

Quantification of inputs and expenditure on specific health systems components, and efforts by us and by the Global Fund [4] to track funds to the district and facility level, were unsuccessful. Therefore, establishment of a causal chain and reliable attribution of health systems effects to particular GHIs is not possible. However, our district level findings do provide empirical evidence that supports other mainly national level studies and government and Ministries of Health reports of increasing workload for health staff, especially in rural areas. Malawi appears to have been somewhat more successful than Zambia in recruiting clinical staff, and more so in allocating HSAs and counsellors to supporting scale up. Despite Zambia's efforts and donor support to its rural health worker incentive and retention scheme [18], progress in implementing its human resources strategic plan has been slow and postings have favoured urban areas at the expense of rural areas [17,23]. The scheme has had limited success due to accommodation shortages, a short timeframe for retention allowances and eligibility criteria that until 2007 included only doctors, though it has since been extended to include nurses and nurse tutors [23]. According to the Ministry of Health in 2009, the current staff establishment contained 32,688 approved positions, though not necessarily funded posts, representing 65% of the staffing requirements for the new structure [31]. Zambia's national Human Resources for Health Strategic Plan [18] has also lacked concerted GHI-support for hiring new health workers [31].

Two explanations may account for the overall less effective scale-up in clinical staff in Zambia: the country may have produced additional clinical staff over 2004-07, but was losing them to better funded posts in the NGO and private for profit sectors (and to emigration) [32], or it was not producing sufficient clinical staff to meet replacement needs. Others have commented on how rural-to-urban staff migration is compounded by public-to-private provider brain drain, as part of a broader phenomenon of rural-urban inequity [33]. Key informant interviews in our study reported that urban facilities in Zambia had benefited more than rural facilities from large levels of new resources; and they also reported significant migration from government employment to well funded NGOs, which we could not confirm and quantify. Two studies have reported that the higher wages offered by PEPFAR-funded NGOs were attracting staff away from the public sector [22,34]. Up to 2007, PEPFAR was paying salary top-ups and overtime payment for ART delivery [34]. Together, these findings suggest a PEPFAR-effect that was benefiting the facilities it supports at the expense of other facilities. Prior to the GHIs becoming major players, NGOs were reported to be paying between 23% and 46% more than government [35]. As Dussault and Franchescini have reported, even where countries have comprehensive health worker policies and strategies, funding may not follow and geographical imbalances result: "Highly-skilled professionals and institutions respond more to incentives than to control mechanisms" [33].

Malawi's health workforce response suggests differences to Zambia in GHI health systems' effects. Support from donors in April 2005 [11], including the Global Fund which agreed to the re-allocation of Malawi's Round 1 grant, enabled Malawi to start to implement its Emergency Human Resource Programme [12]. Demand-side differences, whereby Malawi exerted pressure on the Fund, or supply-side differences, whereby Global Fund portfolio managers interpreted the Fund's guidelines differently in Malawi, could have accounted for this decision to re-allocate the Round 1 grant. As a result, Malawi's Programme has focused on funding basic training (doubling the number of nurses and tripling the number of doctors in training), staff recruitment, deployment (including to rural areas), retention (partly through salary top-ups), basic training and retraining of HSAs to deliver HIV services, and incentives for training tutors [11-13]. Malawi, with the support of the Global Fund through a central pooled mechanism, has been able to invest a greater proportion of its resources on basic training: "... a 165% increase in pre-service training and 79% increase in post-basic training" [12], compared to Zambia.

## Conclusions

The importance of these findings is that they represent what the Global Fund Five Year Evaluation was unable to demonstrate - facility level scale up in clients and service episodes, associating these with indicators of health systems capacity - in this case health worker categories and numbers. The data time-periods are not the same - Malawi's baseline data range from the last quarter of 2005 to early 2008, compared with the start of 2004 to the end of 2007 for Zambia - but clear differences as well as similarities in trends are evident.

### Getting better evidence for action

Our findings illustrate much of the 'messiness' associated with reliance on the data obtained from routine health facility information systems, which health systems in sub-Saharan African countries generate and on which they rely for evidence for action. Routine data that are based on health facility records are prone to errors at all stages from initial recording in facility registers, through compilation of data at the facility level for returns to district health offices, during compilation at the district level for reporting to national level, and in analysis at the national level. Data analysis in this study enabled outliers and data of questionable plausibility to be identified and checked, using original research tools/proformas where available. However, this could not preclude errors earlier in the health information system chain, at the level of the health facility recording and reporting system. Health information performance and problems can also be programme-specific. For example, routine PMTCT data in Malawi was not considered to be reliable up to 2007.

One objective of this paper has been to illustrate the potential from analysing health facility data and our analysis demonstrated some of the methodological problems and responses: median workloads (staff-client ratios) are better measures than means for taking into account changes in smaller facilities with low client numbers, because a small number of facilities with large client numbers can have a disproportionate effect on an analysis that uses means, but both measures are important. The collection of facility level data on trends in this study, which the Global Fund Five Year Evaluation did not attempt, demonstrated how health facilities in Malawi and Zambia have been managing to deliver HIV and AIDS services to much greater numbers, while coping with routine workload. The key informant interview data corroborated and helped to illustrate the effects - and the potential for burnout among health workers. The findings are also consistent with and reinforce other findings on rural-urban inequities in Zambia, particularly in terms of workload. Considerable effort was invested by researchers in Zambia to obtain complete data-sets directly from facilities at baseline (2006-07) and again at follow-up (2008) using improved tools. The objective was to show trends in facility outputs of interest: numbers of HIV and non-HIV clients and service episodes. Similar data were collected from national programme offices in Malawi.

In mid-2008, data sets recording OPD and non-HIV priority service clients and episodes were obtained in electronic format directly from district health management offices in Zambia. Reasons for greater completeness of district records, where this was found, were that many health facilities kept no copies of the returns they had sent to district offices; and some, over-time, discarded or mislaid original records. District health offices in Zambia were more consistent than facilities in recording catchment populations (numbers of adults, under ones and under five year old children, women of child bearing age), which facilitated calculation of coverage rates, including immunisation and family planning coverage (data not shown).

The value of staff-population density calculations is more limited in areas where there is a mixture of government and non-government (for-profit and non-profit) providers, and where there are tertiary specialist hospitals that attract patients from afar. Both of these features are characteristic of urban areas. Where staff density data are more useful is to demonstrate health worker allocations and policy responses in rural districts, as in the case of rural Mumbwa district in Zambia where staff densities were falling. The data in this study do not definitely show a growing health worker density gap between rural and urban facilities, but they point to such a gap in those facilities providing HIV service that had catchment population data. Even in the absence of data from non-public facilities, as was the case in Malawi, the available data can still be translated into evidence that should be available to government, with respect to staff allocations to public sector facilities, and to assist with implementation of the WHO rural retention guidelines and policy recommendations [36].

### Acting on the evidence

Staff retention is not only about salaries, top-ups and financial incentives and includes motivational factors that stem from having the infrastructure, management systems, drugs and other commodities for delivering services [37], which the GHIs have supported. The Global Fund was contributing an estimated 23% of its funding to human resources, though mostly (apart from Malawi) on improving the capacity of existing staff rather than on training and hiring new staff [19]. Malawi's receipt of large levels of resources from only one GHI - the Global Fund, which was aligning itself with government and pooling its funding with other donors and government - may have made it easier for government to roll out a coordinated national health workforce strategy. The training of new clinical staff, which started in 2005-06 in Malawi, would take time; and the training of volunteers and HSAs as HIV counsellors has been a useful quick response [38]. However, task-shifting and short-term in-service training should not be considered panaceas [39] and need to be part of comprehensive government-led strategies [40]. An even greater investment by donors and governments in the basic pre-service training of nurses, clinical officers, medical assistants and doctors is required. It is shortages and lower densities of clinical staff that lead to higher maternal, infant and under-five mortality rates [41].

Up to 2007, PEPFAR had a limit of $1 million per-country to be spent on pre-service training, which was raised to $6 million (or 3% of country budgets) from 2009 [34]. A limited pool of health workers provokes an inevitable competitive tension between programmes funded by government and different donors, especially where GHIs can fund higher salaries and incentives. Reports have highlighted to PEPFAR its lack of support for the production of new health workers and its effects on health worker distribution [31]. The 2008 PEPFAR reauthorisation promised to take the bold step of training 'at least 140,000 new healthcare workers in HIV/AIDS prevention, treatment and care' [42], by 2013, with an initial phase (2009-2010) of identifying opportunities for joint health worker training with GHIs [10]. This may form part of the health systems strengthening component of the new US Global Health Initiative [43]. If overall levels of GHI funding to countries such as Zambia 'flat-line' or decrease [44,45], decisions around the use of available funds to produce and retain new clinical staff, as the Global Fund has enabled to happen in Malawi, will become even more important.

## Competing interests

The authors declare that they have no competing interests.

## Authors' contributions

RB led on study design, data analysis, and drafting of the article. JK participated in study design, data analysis (particularly the Malawi data) and drafting of the article. JS participated in data collection, data analysis (particularly the Zambia data) and drafting of the article. PD participated in data analysis and drafting of the article. VM participated in study design, data analysis (particularly the Malawi data) and drafting of the article). AW participated in data collection, data analysis and drafting of the article. All authors read and approved the final manuscript.
